# Insanity Cured by Influenza

**Published:** 1890-07-05

**Authors:** 


### INSANITY CURED BY INFLUENZA.

In a recent number of the *Neurologisches Centralblatt*, Dr. Metz records the case of a man, aged thirty, who had suffered from mental disease since the summer of 1888. In February, 1889, he became affected with delirium of persecution, and was so violent that he had to be placed in confinement. At the beginning of the present year there was an outbreak of influenza in the asylum. The patient in question was attacked on January 13th; on the 17th he wrote a rational letter to his wife, giving a clear and coherent account of all that had occurred since he had become insane. He dated the recovery of his mental lucidity from the day on which he was attacked with influenza.

